# Iguratimod represses B cell terminal differentiation linked with the inhibition of PKC/EGR1 axis

**DOI:** 10.1186/s13075-019-1874-2

**Published:** 2019-04-11

**Authors:** Yan Ye, Mei Liu, Longhai Tang, Fang Du, Yuanhua Liu, Pei Hao, Qiong Fu, Qiang Guo, Qingran Yan, Xiaoming Zhang, Chunde Bao

**Affiliations:** 1 Department of Rheumatology, Renji Hospital, School of Medicine, Shanghai Jiaotong University, 145 Shandong C Rd, Shanghai, 200001 China; 2 Key Laboratory of Molecular Virology & Immunology, Institut Pasteur of Shanghai, Chinese Academy of Sciences, 320 Yueyang Rd, Shanghai, 200031 China; 3 Suzhou Blood Center, Suzhou, 215006 China

**Keywords:** Iguratimod, Rheumatoid arthritis (RA), Protein kinase C (PKC), Early growth response 1 (*EGR1*), Antibody-secreting cell (ASC)

## Abstract

**Background:**

This study aimed to explore the molecular mechanism and clinical relevance of iguratimod in the regulation of human B cell terminal differentiation.

**Methods:**

An in vitro human antibody-secreting cell (ASC) differentiation system was established to test the effect of iguratimod. B cell phenotype and key transcription factors (TFs) relevant to ASC differentiation were analyzed through flow cytometry and qPCR. The COX-2 activity was measured by enzyme immunoassay (EIA). RNA sequencing was used to identify potential targets of iguratimod. We enrolled six treatment-naive rheumatoid arthritis (RA) patients whose blood samples were collected for phenotypic and molecular studies along with 12-week iguratimod monotherapy.

**Results:**

Iguratimod inhibited human ASC generation without affecting B cell activation and proliferation. Iguratimod showed only weak COX-2 activity. Gene set enrichment analysis (GSEA) identified that protein kinase C (PKC) pathway was targeted by iguratimod which was confirmed by PKC activity detection. Furthermore, early growth response 1 (*EGR1*), a target of PKC and a non-redundant TF for ASC differentiation, was found to be the most downregulated gene in iguratimod-treated B cells. Lastly, iguratimod monotherapy decreased peripheral ASCs and was associated with improved disease activity. The expression of major ASC-related TFs, including *EGR1*, was similarly downregulated in patient blood samples.

**Conclusions:**

Iguratimod inhibits ASC differentiation both in vitro and in RA patients. Our study suggests that PKC/EGR1 axis, rather than COX-2, is critically involved in the inhibitory effect by iguratimod on human ASC differentiation. Iguratimod could have a broader application to treat B cell-related autoimmune diseases in clinics.

**Electronic supplementary material:**

The online version of this article (10.1186/s13075-019-1874-2) contains supplementary material, which is available to authorized users.

## Background

Breaking of B cell tolerance and the generation of high-affinity autoantibodies play a pivotal role in the pathogenesis of autoimmune diseases, including systemic lupus erythematosus (SLE) [1] and rheumatoid arthritis (RA) [2]. Thus, B cell-targeting therapies have attracted great attentions in recent years. B cell depletion by rituximab has succeeded in trials on rheumatoid arthritis (RA) [3–5]; however, its effects on SLE were variable [6, 7]. Other trials to target B cell-activating factor (BAFF) by belimumab and atacicept have been performed on SLE, while only belimumab has achieved positive endpoints [8, 9]. Attempts to arrest B cell activation such as by Bruton tyrosine kinase (Btk) inhibitor ibrutinib are still at a pre-clinical stage [10].

Recently, targeting plasma cells by proteasome inhibitor bortezomib [11, 12] represents a new strategy to remove long-lived plasma cells in autoimmune diseases. Actually, it is less effective for plasma cell depletion in human than in mice. In addition, long-term treatment with bortezomib or other proteasome inhibitors is not favored because of the toxicity profiles and costs [13]. Therefore, new therapies to ablate aberrant B cell compartment with minimum side effects in autoimmune diseases are urgently warranted.

Iguratimod is a conventional synthetic disease-modifying antirheumatic drug (DMARD) approved for treating RA in northeast Asia [14]. In a phase 3 clinical trial performed in Japan, iguratimod showed superiority over placebo and non-inferiority to salazosulfapyridine (SASP) [15]. We have performed the phase III clinical trial in China and found that iguratimod was non-inferior to methotrexate (MTX) with fewer and milder side effects [16]. A postmarketing surveillance (PMS) study involving more than 2000 patients for 52 weeks provided the real-world evidence that iguratimod was safe and effective in RA patients [17]. In addition, iguratimod showed add-on efficacy in RA patients with inadequate response to methotrexate [18], methotrexate-cyclosporin A-hydroxychloroquine-prednisone [19] or biological DMARDs [20, 21].

While the therapeutic benefit of iguratimod is obvious in clinics, currently its mechanism of action is still not fully clarified. Iguratimod was initially developed as a cyclooxygenase-2 (COX-2) inhibitor [22]; however, the mechanism of action of iguratimod seems distinct from classical non-steroidal anti-inflammatory drugs (NSAIDs) [22, 23]. Iguratimod was also shown to inhibit NF-κB translocation [24] and suppress proinflammatory cytokine production in a variety of cell types [24–26]. By contrast, a recent study indicated that iguratimod exhibited minimal suppression for several proinflammatory cytokines (TNF-alpha, MCP-1 and IL-8) in LPS-stimulated human monocytes; instead, it was identified as a macrophage migration inhibitory factor (MIF) inhibitor to exert an anti-inflammatory effect [27]. Iguratimod was also shown to dampen IL-17 signaling by targeting Act1 [28]. All of these studies suggest that iguratimod could play a complex role in vivo and to identify the key pathway for its action is urgently warranted.

In our phase III clinical trial of treatment of active RA with iguratimod, one striking feature closely associated with clinical improvement following iguratimod treatment in RA patients is the reduction of serum concentrations of immunoglobulins such as IgG, IgM, and IgA [15, 16]. In RA and lupus models, iguratimod also decreased autoantibody titers, including anti-collagen [28, 29] and anti-dsDNA [30]. Interestingly, iguratimod seems to regulate B cell differentiation in a unique, non-antiproliferative manner. Unlike B cell depletion agent rituximab [31, 32] or Btk inhibitor ibrutinib [33], iguratimod reduced the peripheral ASC population without affecting other B cell compartments in MRL/lpr mice [30]. Besides, either proliferation or apoptosis of B cells seemed not affected by iguratimod in mice or in vitro [28, 30, 34]. In this study, we have investigated the effect of iguratimod on human B cell terminal differentiation with a purpose to reveal the key pathway targeted by this promising antirheumatic drug.

## Methods

### Patients and treatment

Peripheral blood samples from six new-onset RA patients who fulfilled the ACR/EULAR 2010 criteria were obtained from Renji Hospital, Shanghai, China. Buffy coats were obtained from healthy donors from Blood Center of Changhai Hospital, Shanghai, China. In the study, we also collected peripheral blood samples from six disease-modifying drug-naive RA patients every 4 weeks after receiving iguratimod monotherapy (50 mg/day) until the 12th week. Iguratimod was kindly provided by Simcere Pharmaceutical Group (Nanjing, China). Additional file 1: Table S1 summarized the baseline characteristics of the six patients. Disease activity is calculated by DAS28-CRP, DAS28-ESR, simplified disease activity index (SDAI), and CDAI. This study was approved by the Ethics Committee of Renji Hospital, Shanghai, China. Informed consent was obtained from all study participants. All studies were performed in accordance with the Declaration of Helsinki.

### B cell purification and culture

Peripheral blood mononuclear cells (PBMCs) were collected by Lymphoprep (Axis-Shield) density gradient centrifugation of heparinized blood or buffy coats. Human B lymphocytes were purified from healthy donors using LS Columns and MACS Technology (Miltenyi Biotec). The purity of B cells was checked by flow cytometry (FCM), and the purity was > 95%. CD19+ cells were differentiated into plasma cell in RPMI-1640 medium (GIBCO, North Andover, MA) supplemented with 10 ng/mL IL-2 (Peprotech), 10 ng/mL IL-10 (R&D), 5 μg/ml CpG2006 (Sangon Biotech, Shanghai), 10% fetal bovine serum (FBS) (GIBCO), and antibiotics (penicillin 100 U/ml, streptomycin 100 μg/ml, Gibco BRL). In some experiments, B cells were stimulated with 10 ng/mL IL-2 (Peprotech), 50 ng/ml IL-21 (Miltenyi), and 250 ng/ml multimeric CD40L (Adipogen). To assess the effects of iguratimod on B cell terminal differentiation, B cells were incubated with various concentrations of iguratimod (1, 3, 10 μM, Simcere Pharmaceutical Group) or vehicle (DMSO, sigma) at the onset of culture. Celecoxib (COX-2 inhibitor, Sigma) was added as a control in given experiments. The cells were harvested on days 2, 3, and 5 and analyzed by FCM. Data were analyzed using FlowJo (Tree Star, USA).

### Flow cytometric analysis

PBMCs were surface stained with fluorochrome-labelled antibodies against CD3 (eBioscience, SK7), CD19 (Biolegend, HIB19), CD20 (BD, 2H7), CD27 (Biolegend, O323), and CD38 (Biolegend, HIT2). Circulating ASC was identified as CD3-CD19+CD20-CD27hiCD38hi. To detect B cell activation and intracellular makers of in vitro differentiated B cells, cells were washed and then stained with Zombie Yellow™ Dye (Biolegend) to eliminate dead cells. CD69 (BD, FN50) and CD25 (Biolegend, M-A251) were used to detect B cell activation. For intracellular staining, cells were fixed, permeabilized, and stained for the detection of intracellular markers including BLIMP1 (Novus, 3h2E8), XBP1 (Biolegend, 143F), PAX5 (Biolegend, 1H9), and pSTAT3 (Y705) (eBioscience, LUVNKLA) using a Foxp3 transcription factor staining buffer kit following the manufacturer’s instructions (eBioscience).

### IgG and IgM production assessment

The supernatants were harvested and tested for the concentrations of IgM and IgG by using enzyme-linked immunosorbent assay (ELISA) kits (R&D system, Minneapolis, MN, and ICL Lab) according to the manufacturer’s instructions. The absorbance was read at 450 nm on a microplate reader.

### Flow cytometric analysis of B cell apoptosis and proliferation

Following 48-h culture of B cells, apoptosis was assessed with an apoptosis detection kit with Annexin V/Propidium Iodide (PI), according to the manufacturer’s instructions (eBiosciences). Proliferation was assessed by carboxyfluorescein succinimidyl ester (CFSE) (Sigma) until day 5.

### Real-time quantitative polymerase chain reaction (PCR) analysis

Total RNA from B cells was isolated with TRIzol reagent (Life Technologies) on day 4 and reverse-transcribed using Sensiscript II reverse transcriptase Kit (Takara). mRNA expression of *BLIMP1*, *XBP1*, *PAX5*, *and EGR1* were measured by SYBR Green qPCR using Premix Ex Taq (Takara). Thermocycler conditions included an initial incubation at 95 °C for 15 s. This was followed by a two-step PCR program: 95 °C for 5 s and 60 °C for 30 s for 40 cycles. Each reaction was performed in triplicate. Data were collected and quantitatively analyzed on an ABI PRISM 7900 sequence detection system (Applied Biosystems, Grand Island, NY, USA). The GAPDH gene was used as an endogenous control.

### Enzyme immunoassay(EIA)for COX-2 activity

For COX-2 activity assessment, we used an ex vivo COX-2 inhibitor screening assay kit (No. 701080; Cayman Chemical, USA). In general, COX-2 catalyzes the first step in the biosynthesis of arachidonic acid to prostaglandin H2 (PGH2); then PGH2 was reduced into PGF2α with stannous chloride, which was measured by EIA. DMSO-dissolved iguratimod (1 μM to 1 nM) or celecoxib (1 μM) was applied in the first reaction of this kit.

### Western blotting for EGR1

Following 0, 1, 2, and 4 days of B cell culture, proteins were extracted in lysis buffer (50 mM Tris, pH 7.4; 150 mM NaCl; 1% Triton X-100; and 1 mM EDTA, pH 8.0) supplemented with protease inhibitor complete mini (Roche) and 1 mM PMSF, 1 mM Na3VO4, and 1 mM NaF. The proteins were then separated by SDS-PAGE and electrophoretically transferred onto polyvinylidene fluoride membranes. The membranes were probed with anti-EGR1 mAb (Cell Signaling Technology) overnight at 4 °C and then incubated with an HRP-coupled secondary Ab. Detection was performed using a LumiGLO chemiluminescent substrate system.

### PKC kinase activity assessment

Purified B cell were harvested on 30 min and then lysed to obtain whole cell lysate. PKC kinase activity was detected with a commercial kit (Abcam) and performed according to the manufacturer’s instructions. Measured optical density was at 450 nm.

### RNA-seq analysis

Library preparation for transcriptome sequencing: all RNA-seq experiments were performed with purified B cells after 4 days of culture. Briefly, mRNA was purified from total RNA using poly-T oligo-attached magnetic beads. Fragmentation was carried out using divalent cations under elevated temperature in NEBNext First Strand Synthesis Reaction Buffer (5×). First-strand cDNA was synthesized using random hexamer primer and M-MuLV Reverse Transcriptase (RNase H). Second-strand cDNA synthesis was subsequently performed using DNA polymerase I and RNase H. Remaining overhangs were converted into blunt ends via exonuclease/polymerase activities. After adenylation of 3′ ends of DNA fragments, NEBNext Adaptor with hairpin loop structure was ligated to prepare for hybridization. In order to select cDNA fragments of preferentially 150~200 bp in length, the library fragments were purified with AMPure XP system (Beckman Coulter, Beverly, USA). Then 3 μl USER Enzyme (NEB, USA) was used with size-selected, adaptor-ligated cDNA at 37 °C for 15 min followed by 5 min at 95 °C before PCR. Then PCR was performed with Phusion High-Fidelity DNA polymerase, Universal PCR primers, and Index (X) Primer. At last, PCR products were purified (AMPure XP system) and library quality was assessed on the Agilent Bioanalyzer 2100 system.

The clustering of the index-coded samples was performed on a cBot Cluster Generation System using TruSeq PE Cluster Kit v3-cBot-HS (Illumina) according to the manufacturer’s instructions. After cluster generation, the library preparations were sequenced on an Illumina Hiseq platform and 125 bp/150 bp paired-end reads were generated.

Differential expression analysis of two groups was performed using the DESeq2 R package (1.10.1). DESeq2 provide statistical routines for determining differential expression in digital gene expression data using a model based on the negative binomial distribution. The resulting *P* values were adjusted using the Benjamini and Hochberg’s approach for controlling the false discovery rate. Genes with an adjusted *P* value < 0.05 found by DESeq2 were assigned as differentially expressed. Principle component analysis (PCA) was implemented with prcomp in R package. Gene Set Enrichment Analysis (GSEA) was performed using GSEA software from Broad Institute [35].

### Statistical analysis

Statistical analysis was performed with the GraphPad Prism 7 software. Statistical significance between two groups was calculated by Student’s *t* test or paired Student’s *t* test; for comparisons of more than two groups, one-way or RM one-way ANOVA with Bonferroni correction for multiple comparisons was used. *P* value < 0.05 was considered significant. The statistical evaluation of the RNA-seq data is described in the section dealing with the RNA-seq analysis.

## Results

### Iguratimod inhibits human ASC differentiation upon either T cell-dependent or T cell-independent stimuli

CD40L [36] and CpG [37–40] represent T cell-dependent and T cell-independent B cell-activating agents, respectively. We first screened and optimized the stimulation protocols in order to efficiently generate ASC from human B cells in vitro. We have identified that a combination of CpG2006, IL-2, and IL-10 resulted in the highest ASC yield after 5-day culture (Additional file 1: Figure S1). Thus, we selected this CpG/IL-2/IL-10 protocol for the following studies.

**Fig. 1:**
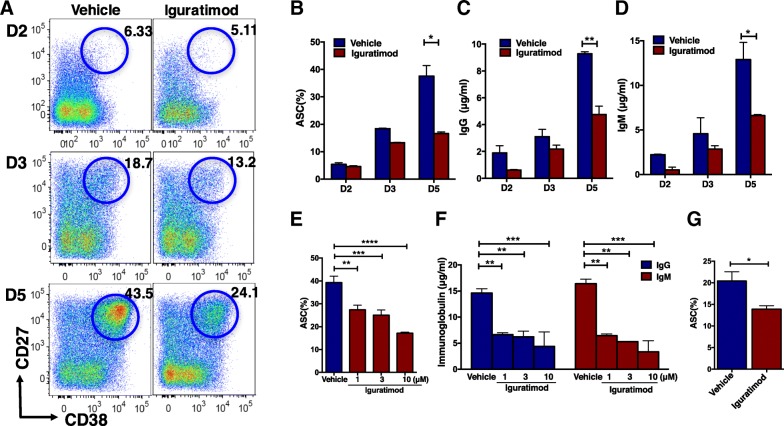
Iguratimod inhibits ASC differentiation in vitro. **a**–**f** Human ASCs were differentiated from B cells by CpG/IL-2/IL-10 in the presence of vehicle (DMSO) or 10 μM iguratimod and checked at day 2, day 3, and day 5. FCM plots (**a**) and cumulative ASC frequencies (**b**, **e**) were shown (*n* = 3). IgG (**c**, **f**) and IgM (**d**, **f**) were detected in the supernatants (*n* = 3). **b**–**f** were detected at day 5 of stimulation in triplicate. One representative of at least three independent experiments was shown (**a**–**f**). **g** Iguratimod inhibited ASC differentiation similarly by CD40L/IL-2/IL-21 (*n* = 5). Data were shown as mean ± SEM and analyzed by Student’s *t* test (**b**–**d**, **f**) or one-way ANOVA with Bonferroni correction for multiple comparisons (**e**, **f**). *P < 0.05, **P < 0.01, ***P < 0.001, ****P < 0.0001

We then tested the effect of iguratimod on the differentiation of human B cells into ASCs. We found that iguratimod at 3 μM inhibited CD27^hi^CD38^hi^ ASC generation and this effect is particularly obvious at day 5 following CpG/IL-2/IL-10 stimulation (Fig. 1a, b). Consequently, the secreted IgG and IgM were also significantly decreased at day 5 in the presence of iguratimod (Fig. 1c, d). Iguratimod inhibited ASC yield and immunoglobulin secretion in a dose-dependent manner, even though the concentration as low as 1 μM was already very potent (Fig. 1e, f). We confirmed the suppressive effect of iguratimod on human ASC generation by CD40L/IL-2/IL-21, a CD40L-based stimulation condition (Fig. 1f). Collectively, these results indicate that iguratimod has a broad inhibitory effect on human ASC generation, irrespective of stimuli.

### Iguratimod does not affect B cell survival, activation, or proliferation

We then tested the effects of iguratimod at a relatively high concentration (10 μM) on human B cell survival and proliferation in the context of CpG/IL-2/IL-10 stimulation. Cell survival was almost intact (Additional file 1: Figure S2A and S2B), as well as activated B cells marked by CD69 and CD25 (Additional file 1: Figure S2C and S2D). B cell proliferation was also not significantly affected by checking CSFE^lo^ proliferated B cells (Additional file 1: Figure S2E and S2F). These results are consistent with early studies performed with mouse B cells [28, 30, 34] and hint that iguratimod may have a unique role to suppress B cell terminal differentiation.

### Iguratimod regulates major transcription factor (TF) network for terminal ASC differentiation

**Fig. 2:**
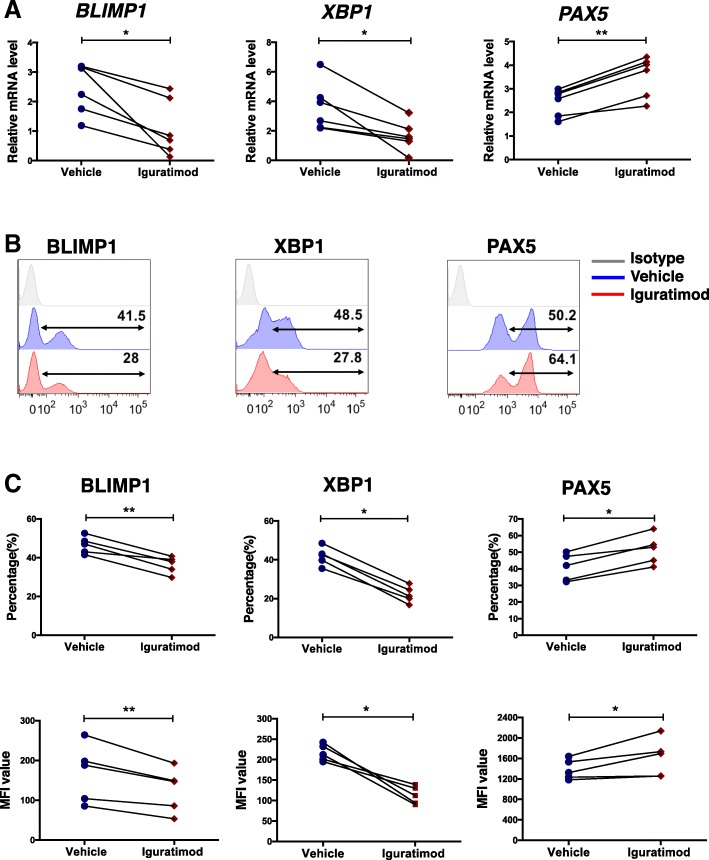
Iguratimod regulates major transcript factor (TF) network of ASC differentiation. Human B cells were stimulated by CpG/IL-2/IL-10 in the presence of vehicle (DMSO) or 10 μM iguratimod and harvested on day 4. **a** Gene expressions of *BLIMP1*, *XBP1*, and *PAX5* were quantified by qRT-PCR (*n* = 6). **b** BLIMP1, XBP1, and PAX5 were measured by flow cytometry, and **c** cumulative data of positive frequencies and median fluorescence intensities (MFI) of individual TFs were also shown (*n* = 5). One representative of at least three independent experiments was shown. Data were analyzed by paired Student’s *t* test (**a** and **c**). *P < 0.05, **P < 0.01

ASC differentiation is coordinated by a set of TFs and particularly BLIMP1, the master TF in this process [41]. We analyzed major TFs for ASC differentiation in stimulated B cells, including BLIMP1, XBP1, and PAX5. Among them, the expressions of BLIMP1 and XBP1 were significantly decreased on the fourth day of stimulation in the presence of iguratimod, while PAX5 was upregulated, both at mRNA (Fig. 2a) and protein (Fig. 2b, c) levels.

Phosphorylation of STAT3 by IL-10 or IL-21 plays a role in ASC differentiation [42]. As IL-10 was used in our stimulation protocol, we checked whether iguratimod affects the phosphorylation status of STAT3. Results showed that iguratimod had no obvious effect on pSTAT3 (Y705) (Additional file 1: Figure S3A, B), indicating that iguratimod does not inhibit ASC differentiation via STAT3 pathway.

### COX-2 is unlikely to be a target of iguratimod in the regulation of ASC differentiation

**Fig. 3:**
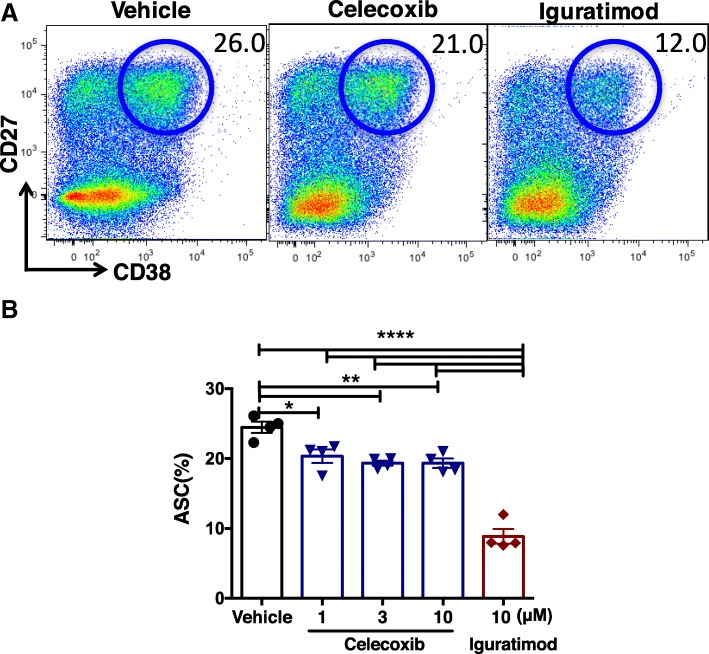
Iguratimod is unlikely to inhibit ASC differentiation via COX-2. Human B cells were stimulated by CpG/IL-2/IL-10 in the presence of vehicle (DMSO), 10 μM celecoxib, or 10 μM iguratimod (**a**) or as indicated (**b**) for 5 days. **a** Representative FCM plots and **b** cumulative data of **a** were shown (*n* = 4). One representative of at least three independent experiments was shown. Data were expressed as mean ± SEM and analyzed by one-way ANOVA with Bonferroni correction for multiple comparisons (**b**). *P < 0.05, **P < 0.01, ****P < 0.0001

There are some reports to indicate that COX-2 could play some role in ASC differentiation [43]. In addition, whether iguratimod can exert its biological function through COX-2 is still controversial. To this end, we tested the idea whether iguratimod could regulate human ASC terminal differentiation through inhibiting COX-2 activity. We chose a typical COX-2 inhibitor celecoxib as a control. We first measured the COX-2 inhibition activities of iguratimod or celecoxib. At the same concentration, iguratimod exhibited a much less COX-2 inhibition activity than celecoxib (Table 1). We then compared the effects of iguratimod and celecoxib on ASC differentiation. Celecoxib showed a very mild inhibitory effect on ASC differentiation ranging from 1 to 10 μM; by contrast, iguratimod showed a much stronger inhibition on ASC yield than celecoxib at 10 μM (Fig. 3a, b). Collectively, these results indicate that COX-2 is an unlikely target of iguratimod to inhibit ASC differentiation.

**Table 1:** Iguratimod only has weak inhibition on COX-2 activity

Celecoxib		Iguratimod	
Concentration (M)	Inhibition (%)	Concentration (M)	Inhibition (%)
1.0 × 10^−6^	97.8	1.0 × 10^−6^	24.9
		1.0 × 10^−7^	19.2
		1.0 × 10^−8^	35.5
		1.0 × 10^−9^	31.3

The COX-2 inhibition activities of iguratimod or celecoxib was determined with cyclooxygenase products, prostaglandins, which were measured by EIA. At the same concentration (1.0 × 10^−6^ M), iguratimod exhibited weak inhibition on COX-2 inhibition activity(24.9%), obviously less than celecoxib (97.8%). One representative experiment out of three was shown

### Iguratimod inhibits PKC activity and EGR1 expression

**Fig. 4:**
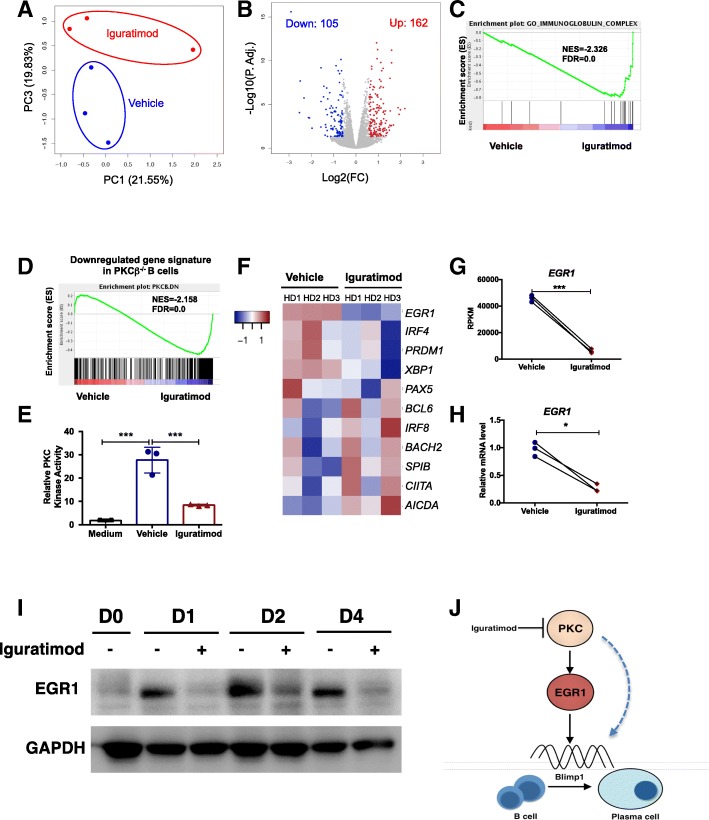
Iguratimod inhibits PKC activity and EGR1 expression. **a**, **b** PCA showed the global gene expression profiles (**a**) and the volcano plot showed the differentially expressed genes (**b**) of the RNA-seq data derived from vehicle (DMSO) or 10 μM iguratimod-treated activated human B cells (*n* = 3). **c**, **d** GSEA identified the immunoglobulin complex pathway (**c**), and PKC pathway (**d**) was inhibited by iguratimod in activated human B cells. **e** PKC activity was decreased upon iguratimod treatment in stimulated human B cells (*n* = 3). **f** Heatmap showed the expressions of key TFs in ASC differentiation and B cell identity from the RNA-seq data derived from vehicle (DMSO) or 10 μM iguratimod-treated activated human B cells (*n* = 3). **g**, **h** *EGR1* expressions were shown by RPKM plots (**g**) or qRT-PCR plots (**h**) (*n* = 3, for **g** and **h**). **i** Western blot showed the suppression of EGR1 in activated human B cells by iguratimod at different time points. One representative experiment out of 3 was shown. **j** A proposed work model for the regulation of plasma cell differentiation by iguratimod via inhibiting PKC and EGR1. Dotted line indicates that PKC may additionally regulate plasma cell differentiation by EGR1-independent pathway. Data were expressed as mean ± SEM (**e**) and analyzed by one-way ANOVA with Bonferroni correction for multiple comparisons (**e**) or paired Student’s *t* test (**g**, **h**). *P < 0.05, ***P < 0.001. RPKM, reads per kilobase of exon per million mapped sequence reads

Next, we used the established human B cell differentiation system to perform a RNA-seq-based gene expression profiling of activated B cells to screen potential targets of iguratimod. Principal component analysis (PCA) indicated that the global gene expression profile of iguratimod-treated samples was different from control samples (Fig. 4a). The volcano plot showed that 162 genes were upregulated while 105 genes were downregulated following iguratimod treatment [adjusted *P* < 0.05, fold change > 1.5] (Fig. 4b). Then we used Gene Set Enrichment Analysis (GSEA) [35] to perform the pathway analysis. We observed a panel of pathways relevant to antibody synthesis were dampened following iguratimod treatment, including immunoglobulin complex (Fig. 4c), responses to endoplasmic reticulum stress, and unfolded protein response (Additional file 1: Figure S4A). While these downstream pathways were well correlated with defective antibody generation effected by iguratimod, we also identified protein kinase C pathway was significantly inhibited by iguratimod (Fig. 4d). Given that PKCβ plays an essential role in plasma cell differentiation [44], these results strongly suggest that iguratimod may target the PKC pathway. We confirmed this hypothesis by showing that iguratimod rapidly inhibited PKC activity in stimulated human B cells (Fig. 4e).

As transcription factors (TFs) play the pivotal role in orchestrating B cell differentiation, we then focused on the B cell- and plasma cell-related TFs and the heatmap indicated that the expression of several plasma-oriented TFs (*IRF4*, *PRDM1* and *XBP1*) was downmodulated and B cell identity TFs (*PAX5, BCL6, IRF8, BACH2, SPIB* and *AICDA*) were increased after iguratimod treatment (Additional file 1: Fig. 4f, Additional file 1: Figure S4B and S4C). Strikingly, we identified that TF early growth receptor 1 (*EGR1*) was greatly downregulated following iguratimod treatment (Fig. 4f, g). EGR1 is a downstream target of PKC [45] and is also a non-redundant TF for BLIMP1 expression [46], raising that EGR1 could be an interesting target. We then confirmed the downregulation of EGR1 expression at the mRNA level by qRT-PCR (Fig. 4h) and at the protein level by western blot (Fig. 4i). Thus, we propose the following scenario that iguratimod suppresses B cell terminal differentiation by inhibiting the PKC pathway and downstream target EGR1. Consequently, the expression BLIMP1 expression is inhibited, resulting in a blockade of plasma cell differentiation (Fig. 4j).

### Iguratimod regulates ASC terminal differentiation in RA patients

**Fig. 5:**
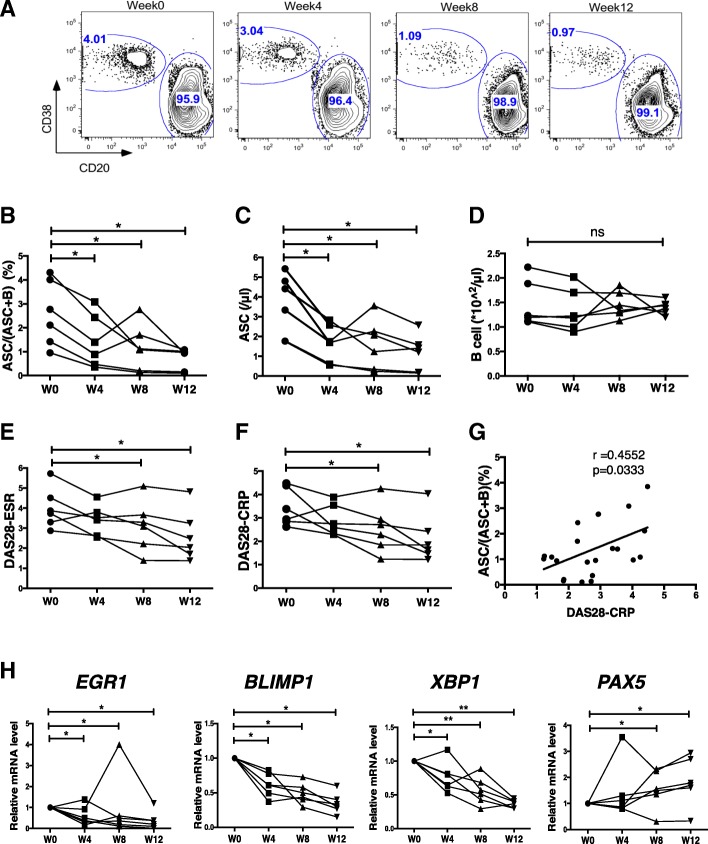
Iguratimod regulates ASC differentiation in naive RA patients. Six naive and active RA patients received iguratimod monotherapy (50 mg/day) for 12 weeks. **a** FCM plots from one representative patient were shown for the changes of peripheral ASC frequencies along the time by the Boolean gating of ASCs and B cells in Flowjo. **b**–**f** ASC frequencies (**b**), ASC numbers (**c**), B cell numbers (**d**), and disease activities calculated by DSR28-ESR and DSR28-CRP (**e** and **f**) were shown before and after treatment (*n* = 6). **g** The correlation between ASC frequencies and DSR28-CRP was shown. **h** *EGR1*, *BLIMP1*, *XBP1*, and *PAX5* mRNAs were detected from PBMC by qRT-PCR (*n* = 6). One representative experiment out of 3 was shown. Data were analyzed with Spearman’s correlation (**g**) or with RM one-way ANOVA with Bonferroni correction for multiple comparisons (**b**–**f**, **h**). *P < 0.05, **P < 0.01, ***P < 0.001, ****P < 0.0001

In our previous randomized controlled trials, we have found that IgG, IgM, and IgA demonstrated a statistically significant decrease after 24 weeks of iguratimod treatment (50 mg/day) [16]. In this study, to validate our findings in clinical practice, we gave six active RA patients iguratimod monotherapy for 12 weeks and collected their blood samples. The range of disease activity calculated by DAS28-CRP was 2.62–4.48. Demographic data of these patients are shown in Additional file 1: Table S1. Consistent with our in vitro findings, peripheral ASC decreased in all patients along with the treatment (Fig. 5a–c), while the total B cell population remained stable (Fig. 5d). All the patients achieved improvements in disease activity (Fig. 5e, f), which was correlated with decrease of peripheral ASC numbers (Fig. 5g). However, we did not find any significant changes of autoantibody titers in such a short period of time, which is consistent with the report that the changes of immunoglobulin will be obvious for a relatively longer time of 24-week treatment [47].

Furthermore, we checked major TF expressions in patients’ peripheral mononuclear cells. Similar to the in vitro studies, the expressions of *BLIMP1*, *XBP1*, and *EGR1* were downregulated while *PAX5* was not altered along with the treatment (Fig. 5h). Collectively, we demonstrate that iguratimod could effectively function in vivo by inhibiting ASC generation and contribute to disease remission in RA patients.

## Discussion

Iguratimod is a new type of DMARDs licensed to treat RA patients in China and Japan. Both clinical trials and clinical practice in large numbers of RA patients provide the convincing evidence that iguratimod is safe and effective with limited adverse effects [15–17]. More importantly, iguratimod provides an additional opportunity for refractory patients to routine first-line and second-line therapies [18–21]. Although the clinical benefit has been proven, the drug target of iguratimod has been controversial, which greatly limits a broader application of this drug. In this study, we uncovered a potential mechanism of iguratimod by demonstrating that iguratimod inhibited human B cell terminal differentiation via dampening PKC pathway and EGR1 expression.

The critical role of B cells and autoantibodies in the pathogenesis of RA has been well appreciated. The abnormal humoral responses indicated by rheumatoid factors (RFs) and anticitrullinated protein antibodies (ACPAs) appear long time before disease onset. Then an attack on the joints happens accompanied with relatively high titers of autoantibodies and with a spread of antibody specificities [48] and activation of osteoclast activity [49]. In addition, B cells reacting with citrullinated peptides were enriched in RA joints [50], supporting a pathogenic role of these B cells locally. A reduction of serum concentrations of immunoglobulins in RA patients following iguratimod treatment suggests that regulating humoral responses is a key aspect of iguratimod [15, 16].

The targets of iguratimod remain controversial, especially for COX-2. In the current study, we showed that targeting COX-2 is an unlike major mechanism by iguratimod in inhibiting ASC differentiation, as iguratimod exhibited a much less COX-2 inhibition activity but more potent to repress ASC generation than a typical COX-2 inhibitor celecoxib. For the other two potential candidates for iguratimod targets, NF-κB [24] and Act1 [28], NF-κB is unlikely to be the target in the current B cell differentiation system, as B cell survival, activation, and proliferation were not affected by iguratimod. Act1 is an adaptor of IL-17 signaling [28] and has also shown to be a negative regulator of CD40- and BAFF-mediated B cell maturation and survival [51, 52]. However, we did not see any significant difference for B cell maturation and survival after iguratimod treatment. Therefore, Act1, as well as NF-κB and COX-2, is not likely to explain effects of iguratimod on B cell terminal differentiation.

To look for potential targets, we applied global RNA sequencing and identified PKC pathway as a new target of iguratimod. The PKC family is broadly divided into three subgroups: classical, novel, and atypical PKCs. In B cells, PKCβ is the most highly expressed PKC member and mediates the signaling downstreams of BCR [53, 54] and BAFF [55]. In a recent report, PKCβ is required for germinal center formation and plasma cell differentiation [44]. In a lupus mouse model, PKCβ deficiency or administration of a PKCβ-specific inhibitor enzastaurin drastically decreases autoantibody titers and prevents the development of the disease [56]. These results demonstrate that PKC pathway, particularly PKCβ, plays a critical role in B cell terminal differentiation.

In addition to PKC pathway, RNA sequencing also identified *EGR1* gene was dramatically downregulated in stimulated B cells following iguratimod treatment. EGR1 is a target of PKC pathway [45] which is a DNA-binding transcriptional regulator important for cell survival, proliferation, and death. The EGR1 binding box was previously demonstrated necessary for BLIMP1 expression [46]. Recently in an in vivo study, *egr1*^−/−^ mice have impaired ASC differentiation yet with normal B cell development and intact cell proliferation and apoptosis [57]. This feature is highly matching what we have observed in iguratimod-treated human B cells. Thus, our study strongly suggests that PKC/EGR1/BLIMP1 axis constitutes a most probable working mechanism for iguratimod to regulate B cell terminal differentiation.

The gene-regulatory network controlling B cell terminal differentiation is complex, and commitment to plasmacytic differentiation involves the inhibition of B cell identity TFs (such as PAX5, BACH2, BCL6 and IRF8) and the expression of the plasma cell-driving TFs (IRF4, BLIMP1 and XBP1) [41]. Particularly, PAX5 and BLIMP1 are identified as two pivotal TFs controlling B cell identity and plasma cell differentiation, respectively, and a mutually antagonistic function of these two TFs has been proposed [58, 59]. In addition to PAX5, BLIMP1 also represses the expression of other B cell identity TFs BCL6, AID, SPIB, and CIITA [60, 61]; thus, an increased expression of these TFs in iguratimod-treated B cells could be linked with the decreased BLIMP1 expression following the PKC/EGR1 axis. In addition, iguratimod treatment also led to enhanced expressions of *BACH2* and *IRF8*. Both BACH2 and IRF8 have been shown to repress BLIMP1 expression, and loss of each promotes ASC differentiation [58, 62]. Interestingly, the expression of both BACH2 and IRF8 is under the control of PAX5 [63], suggesting an indirect effect of decreased expression of BLIMP1. Collectively, we have demonstrated that iguratimod treatment in activated B cells represses ASC differentiation program while promotes B cell program. It is conceivable that the derepression of B cell identity TFs could be via direct or indirect effect of decreased BLIMP1 expression; however, there still could be a possibility that iguratimod may additionally dampen B cell terminal differentiation independent of the PKC/EGR1/BLIMP1 axis, and more studies are needed to elucidate the detailed mechanisms.

EGR1 expression is also strongly increased in both fibroblast-like synovial cell and monocyte from RA patients [64], and cluster analysis shows that EGR1 is one of the top hub TFs in the regulatory network of RA [65]. It will be interesting to see whether iguratimod could inhibit EGR1 expression in other cell types and whether EGR1 could also be a novel treatment target in RA.

## Conclusion

In summary, our study reveals that iguratimod plays a unique role in the regulation of B cell terminal differentiation by targeting PKC and EGR1. While further studies are needed to identify the target molecule directly bound by iguratimod, the current study provides strong support that iguratimod is a promising disease-modifying drug for RA and suggests it may have a broader application in other autoimmune diseases.

## Additional file

Additional file 1: Figure S1. Comparison of different protocols for ASC differentiation in vitro. Human B cells were sorted by CD19 beads from PBMC of healthy donors and stimulated with ten different conditions for 5 days. Frequencies of CD19+CD20-CD27hiCD38hi ASC (A) and immunoglobulins from culture supernatant (B) were shown. (C) FCM plots and (D) cumulative data of ASC generation following CpG/IL-2/IL-10 stimulation at different time points were shown (*n* = 3). One representative of at least three independent experiments was shown. Data were shown as mean ± SEM and analyzed by one-way ANOVA with Bonferroni correction for multiple comparisons (C). *P < 0.05, ***P < 0.001, ****P < 0.0001. Figure S2. Iguratimod does not affect B cell apoptosis, activation, or proliferation. Human B cells were stimulated with CpG/IL-2/IL-10, in the presence of vehicle (DMSO) or 10 μM iguratimod. (A, B) Both apoptotic (Annexin V+PI-) and dead cells (Annexin V+PI+) were not significantly changed with iguratimod treatment at 48 h (*n* = 3). (C) CD69 or CD25 stains were not changed at 48 h. (D) Cumulative data of the MFIs of CD69 and CD25 were shown (*n* = 3). (E, F) Proliferating B cell population (CSFElo) was not significantly changed at day 5 (*n* = 3). One representative of at least three independent experiments was shown. Data were shown as mean ± SEM (B, D, F) and analyzed by Student’s *t* test (B, D) or one-way ANOVA (F). MFI, median fluorescence intensity. Figure S3. Iguratimod does not affect the phosphorylation status of STAT3 in human B cells following IL-21 stimulation. Human B cells were pretreated with vehicle (DMSO) or 10 μM iguratimod for 30 min and then stimulated with IL-21 for 15 min. pSTAT3 (Y705) was detected by flow cytometry. (A) Representative FCM plots and (B) cumulative data of pSTAT3 were shown (*n* = 3). One representative of at least three independent experiments was shown. Data were shown as mean ± SEM and analyzed by one-way ANOVA (B). ***P < 0.001. Figure S4. Iguratimod inhibits TFs required for ASC differentiation in RNA-seq data. (A) GSEA identified the responses to endoplasmic reticulum stress and unfolded protein response pathways were inhibited by iguratimod in activated human B cells. (B, C) RPKM plots showed the expressions of key TFs in ASC differentiation (B) and maintaining B cell identity (C) from the RNA-seq data derived from vehicle (DMSO) or 10 μM iguratimod-treated activated human B cells (*n* = 3). Data were expressed as mean ± SEM and analyzed by paired Student’s *t* test (B, C). *P < 0.05. RPKM, reads per kilobase of exon per million mapped sequence reads. Table S1. Baseline characteristics of the six naive RA patients. Table S2. qRT-PCR primers used in this study (DOCX 9552 kb)

## Abbreviations

ACPA: Anticitrullinated protein antibodies
ASC: Antibody-secreting cell
BAFF: B cell activating factor
Btk: Bruton tyrosine kinase
CFSE: Carboxyfluorescein succinimidyl ester
COX-2: Cyclooxygenase-2
DMARD: Disease-modifying antirheumatic drug
*EGR*: Early growth response 1
EIA: Enzyme immunoassay
ELISA: Enzyme-linked immunosorbent assay
GSEA: Gene set enrichment analysis
MIF: Migration inhibitory factor
MTX: Methotrexate
NSAIDs: Non-steroidal anti-inflammatory drugs
PBMC: Peripheral blood mononuclear cells
PCR: Polymerase chain reaction
PI: Propidium iodide
PKC: Protein kinase C
PMS: Postmarketing surveillance
RA: Rheumatoid arthritis
RF: Rheumatoid factors
SASP: Salazosulfapyridine
SDAI: Simplified disease activity index
SLE: Systemic lupus erythematosus
TF: Transcription factors
