# Supplementary material for: Iguratimod represses B cell terminal differentiation linked with the inhibition of PKC/EGR1 axis
Source: Arthritis Res Ther. 2019 Apr 11;21:92. doi: 10.1186/s13075-019-1874-2 (PMC6458835; doi:10.1186/s13075-019-1874-2)

**Supplementary Materials**

**Figure S1. Comparison of different protocols for ASC differentiation in vitro.** Human B cells were sorted by CD19 beads from PBMC of healthy donors and stimulated with ten different conditions for 5 days. Frequencies of CD19+CD20-CD27hiCD38hi ASC (A) and immunoglobulins from culture supernatant (B) were shown. (C) FCM plots and (D) cumulative data of ASC generation following CpG/IL-2/IL-10 stimulation at different time points were shown (n=3). One representative of at least three independent experiments was shown. Data were shown as mean±SEM and analyzed by one-way ANOVA with Bonferroni correction for multiple comparisons (C). * *P*<0.05, *** *P*<0.001, **** *P*<0.0001.

**Figure S2. Iguratimod does not affect B cell apoptosis, activation or proliferation.** Human B cells were stimulated with CpG/IL-2/IL-10, in the presence of vehicle (DMSO) or 10 μM iguratimod. (A, B) Both apoptotic (Annexin V+PI-) and dead cells (Annexin V+PI+) were not significantly changed with iguratimod treatment at 48h (n=3). (C) CD69 or CD25 stains were not changed at 48h. (D) Cumulative data of the MFIs of CD69 and CD25 were shown (n=3). (E, F) Proliferating B cell population (CSFElo) were not significantly changed at day 5 (n=3). One representative of at least three independent experiments was shown. Data were shown as mean±SEM (B, D, F) and analyzed by Student’s *t* test (B, D) or one-way ANOVA (F). MFI, Median Fluorescence Intensity.

**Figure S3. Iguratimod does not affect the phosphorylation status of STAT3 in human B cells following IL-21 stimulation.** Human B cells were pretreated with vehicle (DMSO) or 10 μM iguratimod for 30 min and then stimulated with IL-21 for 15 min. pSTAT3 (Y705) was detected by flow cytometry. (A) Representative FCM plots and (B) cumulative data of pSTAT3 was shown (n=3). One representative of at least three independent experiments was shown. Data were shown as mean±SEM and analyzed by one-way ANOVA (B). *** *P*<0.001

**Figure S4. Iguratimod inhibits TFs required for ASC differentiation in RNA-seq data.**  (A) GSEA identified the Responses to Endoplasmic Reticulum Stress and Unfolded Protein Response pathways were inhibited by iguratimod in activated human B cells. (B-C) RPKM plots showed the expressions of key TFs in ASC differentiation (B) and maintaining B cell identity (C) from the RNA-seq data derived from vehicle (DMSO) or 10 μM iguratimod-treated activated human B cells (n=3). Data were expressed as mean±SEM and analyzed by paired Student’s *t* test (B,C).

* *P*<0.05. RPKM, reads per kilobase of exon per million mapped sequence reads.

**Table S1. Baseline characteristics of the six naive RA patients**


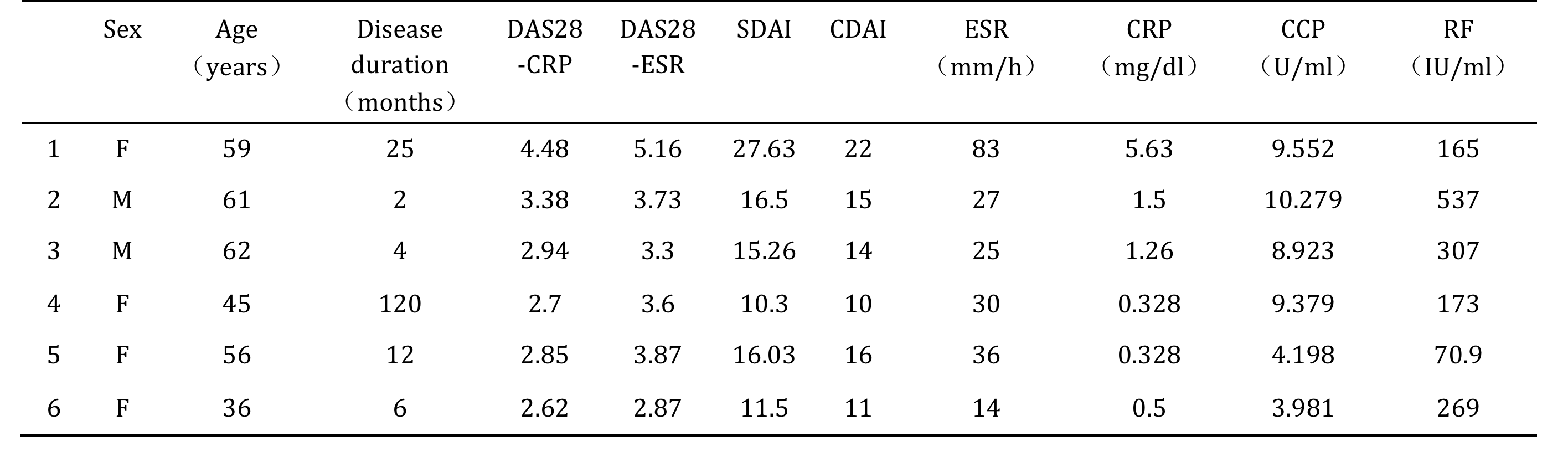


Note: All patients received iguratimod monotherapy (50 mg/d) as their first treatment. Disease activity is calculated by DAS28-CRP, DAS28-ESR, simplified disease activity index (SDAI) and CDAI. Anti-CCP, anti-cyclic citurated peptide antibody; RF, rheumatoid factor.

**Table S2. qRT-PCR primers used in this study**


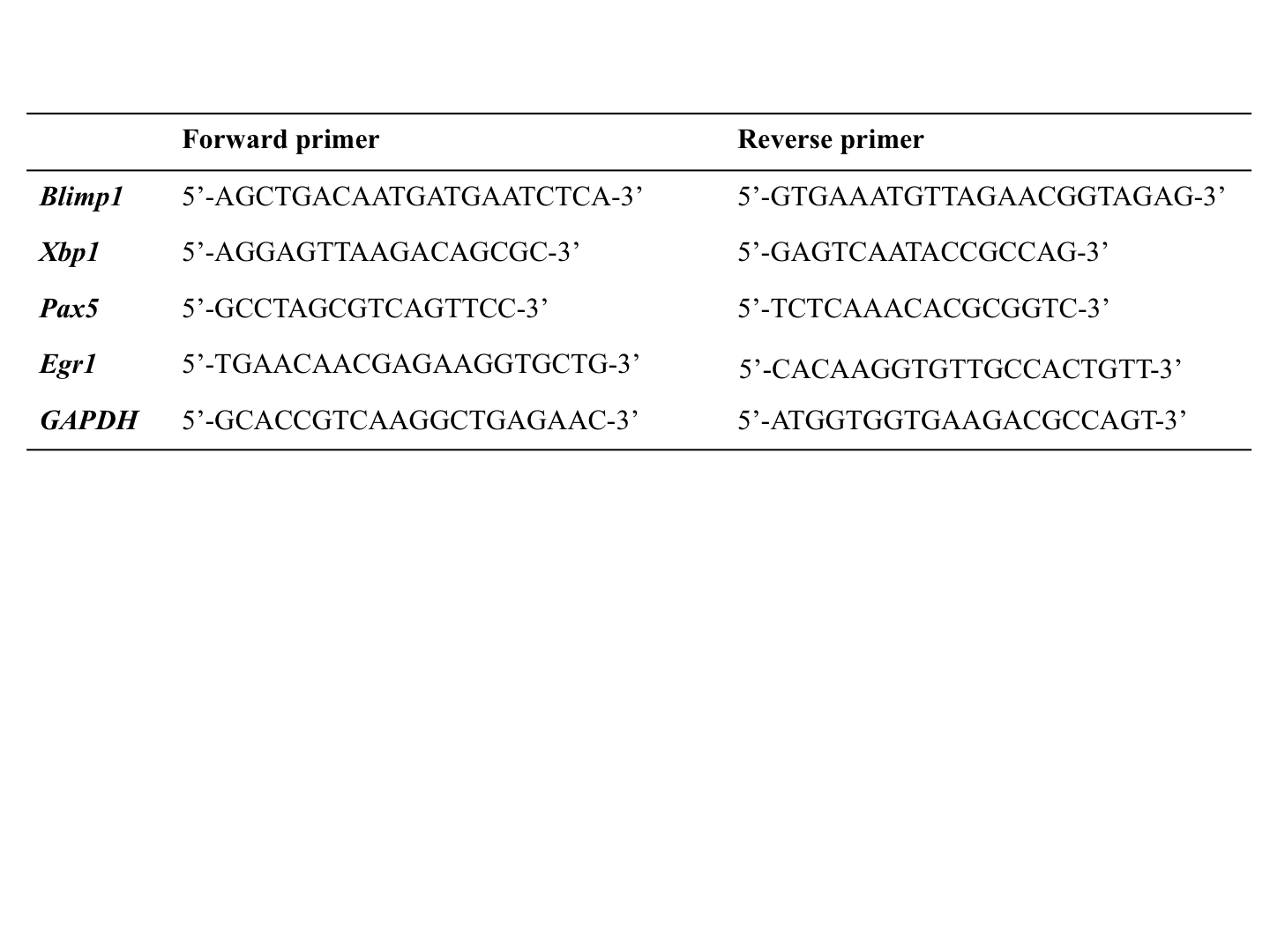

Supplement: Supplementary file 1 — Figure S1. Comparison of different protocols for ASC differentiation in vitro. Human B cells were sorted by CD19 beads from PBMC of healthy donors and stimulated with ten different conditions for 5 days. Frequencies of CD19+CD20-CD27hiCD38hi ASC (A) and immunoglobulins from culture supernatant (B) were shown. (C) FCM plots and (D) cumulative data of ASC generation following CpG/IL-2/IL-10 stimulation at different time points were shown (n = 3). One representative of at least three independent experiments was shown. Data were shown as mean ± SEM and analyzed by one-way ANOVA with Bonferroni correction for multiple comparisons (C). *P < 0.05, ***P < 0.001, ****P < 0.0001. Figure S2. Iguratimod does not affect B cell apoptosis, activation, or proliferation. Human B cells were stimulated with CpG/IL-2/IL-10, in the presence of vehicle (DMSO) or 10 μM iguratimod. (A, B) Both apoptotic (Annexin V+PI-) and dead cells (Annexin V+PI+) were not significantly changed with iguratimod treatment at 48 h (n = 3). (C) CD69 or CD25 stains were not changed at 48 h. (D) Cumulative data of the MFIs of CD69 and CD25 were shown (n = 3). (E, F) Proliferating B cell population (CSFElo) was not significantly changed at day 5 (n = 3). One representative of at least three independent experiments was shown. Data were shown as mean ± SEM (B, D, F) and analyzed by Student’s t test (B, D) or one-way ANOVA (F). MFI, median fluorescence intensity. Figure S3. Iguratimod does not affect the phosphorylation status of STAT3 in human B cells following IL-21 stimulation. Human B cells were pretreated with vehicle (DMSO) or 10 μM iguratimod for 30 min and then stimulated with IL-21 for 15 min. pSTAT3 (Y705) was detected by flow cytometry. (A) Representative FCM plots and (B) cumulative data of pSTAT3 were shown (n = 3). One representative of at least three independent experiments was shown. Data were shown as mean ± SEM and analyzed by one-way ANOVA (B). ***P < 0.001. Figure S4. Iguratimod inhibits TFs re [file 13075_2019_1874_MOESM1_ESM.docx]
